# A Novel Target of IscS in *Escherichia coli:* Participating in DNA Phosphorothioation

**DOI:** 10.1371/journal.pone.0051265

**Published:** 2012-12-11

**Authors:** Xianhui An, Wei Xiong, Yan Yang, Fuhou Li, Xiufen Zhou, Zhijun Wang, Zixin Deng, Jingdan Liang

**Affiliations:** 1 State Key Laboratory of Microbial Metabolism and School of Life Science and Biotechnology, Shanghai Jiao Tong University, Shanghai, People's Republic of China; 2 Ocean College, HuaiHai Institute of Technology, Lianyungang, Jiangsu, People's Republic of China; New England Biolabs, Inc., United States of America

## Abstract

Many bacterial species modify their DNA with the addition of sulfur to phosphate groups, a modification known as DNA phosphorothioation. DndA is known to act as a cysteine desulfurase, catalyzing a key biochemical step in phosphorothioation. However, bioinformatic analysis revealed that 19 out of the 31 known *dnd* gene clusters, contain only four genes (*dndB-E*), lacking a key cysteine desulfurase corresponding gene. There are multiple cysteine desulfurase genes in *Escherichia coli*, but which one of them participates into DNA phosphorothioation is unknown. Here, by employing heterologous expression of the *Salmonella enterica dnd* gene cluster named *dptBCDE* in three *E. coli* mutants, each of which lacked a different cysteine desulfurase gene, we show that IscS is the only cysteine desulfurase that collaborates with *dpt*B-E, resulting in DNA phosphorothioation. Using a bacterial two-hybrid system, protein interactions between IscS and DptC, and IscS and DptE were identified. Our findings revealed IscS as a key participant in DNA phosphorothioation and lay the basis for in-depth analysis of the DNA phosphorothioation biochemical pathway.

## Introduction

Sequence and stereo specific physiological DNA phosphorothioation occurs in many bacteria [1]–[4]. In *Streptomyces lividans* 1326, a five-gene cluster, *dndA–E*, determines the modification [1]. Orthologs of these genes were found in 30 bacterial species and one Archaea [2]. The *dnd* genes are usually located on genomic islands that were probably acquired by horizontal gene transfer [3].

Several of these gene clusters contain *dndB-E* homologues, but lack a *dndA* homologue [2], [3]. In-frame deletion of *dndA* in *S. lividans* showed that the gene is essential for DNA phosphorothioation [1], [4]. DndA was then shown to be a cysteine desulfurase involved in the iron-sulfur cluster assembly for apo-Fe DndC [5].

**Figure 1:**
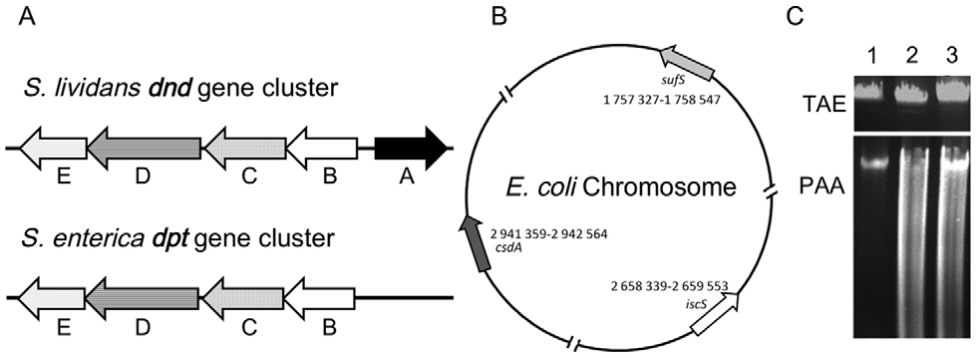
Heterologous expression of the *S. enterica* serovar cerro 87 *dptBCDE* genes in *E. coli* BW25113. A. Orthologous DNA phosphorothioation gene clusters from *S. lividans* (*dndABCDE*) and *S. enterica* (*dptBCDE*). The cysteine desulfurase gene *dndA* of *S. lividans* is required for DNA phosphorothioation. The *S. enterica dptBCDE* gene cluster lacks a dndA ortholog. The *dndA* function may be performed by an unknown, unlinked gene in *S. enterica* and also *in E. coli* expressing *dptBCDE*. B. The three cysteine desulfurases in the *E. coli* genome. C. *E. coli* BW25113 DNA becomes phosphorothioated when expressing *dptBCDE* of S. enterica. Ethidium bromide-stained agarose gels containing total genomic DNA, separated in Tris-acetate EDTA (TAE) buffer. TAE (top panel), untreated samples; PAA (bottom panel), identical DNA samples after incubation in TAE containing 1% per-acetic acid (PAA). Lane 1, *E. coli* BW25113 (wild-type, not S-modified); lane 2, *S. enterica* serovar 87 (wild-type, containing phosphorothioate DNA); lane 3, *E. coli* BW25113 expressing the *S. enterica* serovar cerro 87 *dptBCDE* gene cluster. The fluorescent smear in lanes 2 and 3 of the lower gel indicates that the DNA was phosphorothioate modified.

*Salmonella. enterica* serovar cerro 87 contains *dndB-E* orthologs that are called *dptB-E* [6]. There is, however, no *dndA* ortholog in the entire 20 kb genomic island that contains the *dpt* genes (Fig. 1A) [2]. Heterologous expression of *dptB-E* in *E. coli* DH10B [7] resulted in DNA phosphorothioation [8]. Since DndA is essential for DNA phosphorothioation in *S. lividans*, we hypothesized that there should be one or more genes in the *E. coli* genome that could provide the cysteine desulfurase activity known to be necessary for the modification. Searching for a putative *dndA* orthologue in *E. coli* BW25113 was easier than in *S. enterica* because of the availability of a comprehensive library of knockout mutants of all nonessential genes [9]. In *E.coli*, there are at least three different cysteine desulfurases: IscS, SufS and CsdA [10], . Here we show that only one of them, IscS, supports DNA phosphorothioation in *E. coli* expressing the *S. enterica dptB-E* gene cluster. Protein interactions, which are likely necessary for DNA phosphorothioation, were detected between IscS and both DptC and DptE.

## Materials and Methods

### Bacterial strains, plasmids and primers

Bacterial strains, plasmids, and primers are listed in Table 1, 2 and 3.

**Table 1:** Strains that are used in this study.

STRAINS	CHARACTERISTICS	REFERENCE
*Salmonella enterica* Cerro 87	Strain containing naturally S-modified DNA, source of the *dptB-E* gene cluster	[6]
*E. coli* DH10B	Non-restricting host strain for gene cloning	[7]
*E. coli* BW25113	*acI* ^q^ *rrnB*_T14_ *ΔlacZ*_WJ16_ *hsdR514 ΔaraBAD*_AH33_ *Δ*rhaBAD_LD78_, strain used for creating gene knockouts	[12]
BL21(DE3)pLysS	Lacks lon and ompT proteases Cml^r^	Novagen
JW2514-4	*E.coli* F-, *Δ(araD-araB)567*, *ΔlacZ4787*(::rrnB-3), *&lambda^−^*, *ΔiscS776::kan*, *rph-1*, *Δ(rhaD-rhaB)568*, *hsdR514*	Yale Coli Genetic Stock Center [9]
JW1670-1	*E.coli* F-, *Δ(araD-araB)567*, *ΔlacZ4787*(::rrnB-3), *&lambda^−^*, *ΔsufS755::kan*, *rph-1*, *Δ(rhaD-rhaB)568*, *hsdR514*	Yale Coli Genetic Stock Center [9]
JW2781-1	*E.coli* F-, *Δ(araD-araB)567*, *ΔlacZ4787*(::rrnB-3), *&lambda^−^*, *ΔcsdA738::kan*, *rph-1*, *Δ(rhaD-rhaB)568*, *hsdR514*	Yale Coli Genetic Stock Center [9]
JW2513-1	*E.coli* F-, *Δ(araD-araB)567*, *ΔlacZ4787*(::rrnB-3), *&lambda^−^*, *ΔiscU775::kan*, *rph-1*, *Δ(rhaD-rhaB)568*, *hsdR514*	Yale Coli Genetic Stock Center [9]
JW3955-2	*E.coli* F-, *Δ(araD-araB)567*, *ΔlacZ4787*(::rrnB-3), *&lambda^−^*, *rph-1*, *Δ(rhaD-rhaB)568*, *ΔthiS762::kan*, *hsdR514*	Yale Coli Genetic Stock Center [9]
JW3956-1	*E.coli* F-, *Δ(araD-araB)567*, *ΔlacZ4787*(::rrnB-3), *&lambda^−^*, *rph-1*, *Δ(rhaD-rhaB)568*, *ΔthiF763::kan*, *hsdR514*	Yale Coli Genetic Stock Center [9]
JW2512-1	*E.coli* F-, *Δ(araD-araB)567*, *ΔlacZ4787*(::rrnB-3), *&lambda^−^*, *ΔiscA774::kan*, *rph-1*, *Δ(rhaD-rhaB)568*, *hsdR514*	Yale Coli Genetic Stock Center [9]
JW2508-1	*E.coli* F-, *Δ(araD-araB)567*, *ΔlacZ4787*(::rrnB-3), *&lambda^−^*, *ΔiscX770::kan*, *rph-1*, *Δ(rhaD-rhaB)568*, *hsdR514*	Yale Coli Genetic Stock Center [9]
JW0810-2	*E.coli* F-, *Δ(araD-araB)567*, *ΔlacZ4787*(::rrnB-3), *&lambda^−^*, *ΔmoeB726::kan*, *rph-1*, *Δ(rhaD-rhaB)568*, *hsdR514*	Yale Coli Genetic Stock Center [9]
JW3779-3	*E.coli* F-, *Δ(araD-araB)567*, *ΔlacZ4787*(::rrnB-3), *&lambda^−^*, *rph-1*, *ΔcyaY752::kan*, *Δ(rhaD-rhaB)568*, *hsdR514*	Yale Coli Genetic Stock Center [9]
JW3435-1	*E.coli* F-, *Δ(araD-araB)567*, *ΔlacZ4787*(::rrnB-3), *&lambda^−^*, *ΔyhhP(tusA)725::kan*, *rph-1*, *Δ(rhaD-rhaB)568*, *hsdR514*	Yale Coli Genetic Stock Center [9]
JW0413-1	*E.coli* F-, *Δ(araD-araB)567*, *ΔlacZ4787(::rrnB-3)*, *ΔthiI780::kan*, *λ-*, *rph-1*, *Δ(rhaD-rhaB)568*, *hsdR514*	Yale Coli Genetic Stock Center [9]
AXH034	*E.coli* F-, *Δ(araD-araB)567*, *ΔlacZ4787*(::rrnB-3), *&lambda^−^*, *ΔiscS1191::kan*, *rph-1*, *Δ(rhaD-rhaB)568*, *hsdR514*	This study
*E. coli* XL1-Blue MR	Host strain for propagating pBT and pTRG recombinants Δ(*mcrA*)*183* Δ(*mcrCB-hsdSMR-mrr*)*173 endA1 supE44 thi-1 recA1 gyrA96; relA1 lac*	BacterioMatch II Kit (Agilent)
*E. coli* XL1-Blue MRF′ Kan	Derivative of XL1-Blue MR. Reporter strain for two-hybird test using pBT and pTRG derivatives *Δ*(*mcrA*)183 *Δ*(*mcrCB-hsdSMR-mrr*)173 *endA1 supE44 thi-1 recA1 gyrA96 relA1 lac* [F′ *proAB lacI* ^q^ *ZΔM15* Tn5 (*Kan* ^r^)]	BacterioMatch II Kit (Agilent)

**Table 2:** Plasmids that are used in this study.

PLASMIDS	CHARACTERISTICS	REFERENCE
pKD46	*amp rep^ts^* (30° for replication, 42° for curing)	[7]
pJTU3510	*dptB-E* from *S. enterica* Cerro 87, p15A origin of replication, Cml^r^	This study
pJTU3523	*dptC* from *S. enterica* Cerro 87, cloned in pSJ7 expression vector	This study
pJTU3525	*dptE* from *S. enterica* Cerro 87, cloned in pSJ7 expression vector	This study
pBT	Bait plasmid, λcI Cml^r^, cloning between *Not*I and *Xho*I	bacterioMatch II Two-Hybrid System Vector Kit (Agilent)
pTRG	Target plasmid, Tet^r^, cloning between *Bam*HI and *Xho*I	bacterioMatch II Two-Hybrid System Vector Kit (Agilent)
pBT-LGF2	Control plasmid λcI LGF2 Cml^r^	bacterioMatch II Two-Hybrid System Vector Kit (Agilent)
pTRG-GAL11P	Control plasmid RNAP-α GAL11P^r^	bacterioMatch II Two-Hybrid System Vector Kit (Agilent)
pJTU3609	*dptB* cloned in pTRG with site *Bam*HI and *Xho*I	This study
pJTU3610	*dptC* cloned in pTRG with site *Bam*HI and *Xho*I	This study
pJTU3611	*dptD*cloned in pTRG with site *Bam*HI and *Xho*I	This study
pJTU3612	*dptE* cloned in pTRG with site *Bam*HI and *Xho*I	This study
pJTU3618	*iscS* cloned in pBT with site *Not*I and *Xho*I	This study
pET15b	Expression vector with His_6_-tag Amp^r^	Novagen
pJTU3619	Expressing *E. coli iscS* (amplified using primers *iscS* exU/exD) in pET15b *Nde*I and *Bam*HI	This study
pJTU3625	pJTU3619 derivative site mutant with C111A	This study
pJTU3626	pJTU3619 derivative site mutant with C170A	This study
pJTU3627	pJTU3619 derivative site mutant with C328A	This study
pJTU3622	*dptC* with TEV site insert into pGEX-6P-1 between *Sma*I and *Xho*I	This study
pJTU3624	*dptE* with TEV site insert into pGEX-6P-1 between *Sma*I and *Xho*I	This study

**Table 3:** Primers that are used in this study.

PRIMERS	SEQUENCE	USE
P1	ATTCCGGGGATCCGTCGACC	Amplification of neo FRT
P2	TGTAGGCTGGAGCTGCTTC	Amplification of neo FRT
H1P1	GGTAGCCTGATTCCTTGCATTGAGTGATGTACGGAGTTTATAGAGCAATGATTCCGGGGATCCGTCGACC	Replacement of *iscS*
H2P2	ATTATAAATTCTCCTGATTCCGATACCGATTAATGATGAGCCCATTCGATTGTAGGCTGGAGCTGCTTC	Replacement of *iscS*
U	AAGTGCTGGATGTGTCTG	Verification of *iscS* deletion
D	GACGTTCTCGTCGTTGTT	Verification of *iscS* deletion
iscS exU	GGGAATTCCATATGAAATTACCGATTTATC	To clone *iscS* with *Nde*I site
*iscS* exD	CCGGGATCCAGCCATTATAAATTCTCC	To clone *iscS* with *Bam*HI site
GST-*dptC* F	TGATTACGATATCCCAACGAC	To clone *dptC*
GST-*dptC* R	CCGCTCGAGTGTAATACCAGTTG	To clone *dptC* with *Xho*I site
GST-*dptE* F	TGATTACGATATCCCAACGAC	To clone *dptE*
GST-*dptE* R	CCGCTCGAGGTTGATGCTGCCGT	To clone *dptC* with *Xho*I site
C111A F	GCGGTACTGGATACCGCACGTCAGCTGGAGCGC	Mutated site in IscS
C111A R	GCGCTCCAGCTGACGTGCGGTATCCAGTACCGC	Mutated site in IscS
C170A F	GCTATCGGCGAAATGGCACGTGCTCGTGGCATT	Mutated site in IscS
C170A R	AATGCCACGAGCACGTGCCATTTCGCCGATAGC	Mutated site in IscS
C328A F	TCTTCAGGTTCCGCCGCAACGTCAGCAAGCCTC	Mutated site in IscS
C328A R	GAGGCTTGCTGACGTTGCGGCGGAACCTGAAGA	Mutated site in IscS
IscS-CMu F	ATCTGACAACCTGGCGATCA	To verify IscS mutantions
IscS-CMu R	CTTCAGTAGTAAAACGACCT	To verify IscS mutantions
*dptB*TRG U	CCGGGATCCATGGCTAGTGTTGATGCAG	To clone *dptB* to pTRG with *Bam*HI
*dptB*TRG D	CCGCTCGAGAAATCGTAGGCCTGAACT	To clone *dptB* to pTRG with *Xho*I
*dptC*TRG U	CCGGGATCCATGAGTAAATTAGTTCAGG	To clone *dptC* to pTRG with *Bam*HI
*dptC*TRG D	CCGCTCGAGTTATGTAATACCAGTTGC	To clone *dptC* to pTRG with *Xho*I
*dptD*TRG U	CCGGGATCCATGCGGGCGAATCGTCTG	To clone *dptD* to pTRG with *Bam*HI
*dptD*TRG D	CCGCTCGAGCCATTCGATTCGGGAGCA	To clone *dptD* to pTRG with *Xho*I
*dptE*TRG U	CCGGGATCCATGCTCCCGAATCGAATG	To clone *dptE*to pTRG with *Bam*HI
*dptE*TRG D	CCGCTCGAGTTGATGCTGCCGTAAAAG	To clone *dptE* to pTRG with *Xho*I
*iscS* BT U	ATAAGAATGCGGCCGCAATGAAATTACCGATTTAT	To clone *iscS* to pBT with *Not*I
*iscS* BT D	CCGCTCGAGCCATTATAAATTCTCC	To clone *iscS* to pBT with *Xho*I

**Figure 2:**
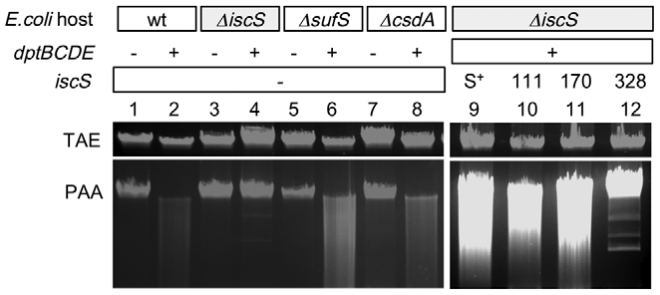
*E.coli iscS* is required for DNA phosphorothioation. Ethidium bromide-stained agarose gels containing *E. coli* total genomic DNA, separated in Tris-acetate EDTA (TAE) buffer. Top gel (TAE), untreated samples; bottom gel (PAA), identical DNA samples after incubation in TAE containing 1% per-acetic acid (PAA). A fluorescent smear in the lower gel indicates that the DNA was S-modified. Lanes 1–8, Dnd (DNA degradation) phenotypes of *E. coli* cysteine desulfurase deletion mutants (*ΔiscS*, *ΔsufS*, *ΔcsdA*) containing the *S. enterica dptBCDE* gene cluster cloned on pJTU3510 (lane 1–8); lanes 9–12, trans complementation of the chromosomal *ΔiscS* mutation by pJTU3619 containing mutant derivatives of *iscS* (lanes 9–12). E. coli hosts: wt, wild type. The mutations *ΔiscS*, *ΔsufS* and *ΔcsdA* are in the *E. coli* chromosome. pJTU3510: −, no plasmid; +, pJTU3510 expressing *dptBCDE*. pJTU3619 (compatible with pJTU3510) containing the following genes: S+, wild-type *E. coli iscS*; 111, 170, 328, mutant *iscS* genes containing the aa changes Cys111Ala, Cys170Ala or Cys328Ala, respectively. −, no plasmid. TAE, gel running buffer; PAA, TAE containing per-acetic acid.

The *E. coli* BW25113 gene replacement mutants listed in Table 1 were obtained from Yale Coli Genetic Stock Center [9]. Among these, the *iscS* mutant JW2514 was not viable, and was recreated by using the gene knockout method described by Datsenko [12]. For this, the neo-FRT (FLP, recombinase recognition target) cassette was amplified using primer P1 and P2, then H1P1 and H2P2. Successful *iscS* deletion was confirmed by PCR using the flanking primers U and D (**Fig. 2A**).

### Detection of DNA phoshorothioation

Phosphorothioate DNA is sensitive to double-strand cleavage by Tris-peracetic acid (TPA) [13]. The phosphorothioation was detected by incubating DNA samples for 30 min at 25°C in TAE buffer (40 mM Tris, 20 mM sodium acetate, 0.8 mM EDTA pH 7.5) supplemented with 1.0% peracetic acid. Phosphorothioate DNA, but not normal DNA, shows Dnd phenotype, producing a smear of DNA fragments in an agarose gel. To prevent DNA degradation during electrophoresis, 50 mM thiourea was added to the TAE electrophoresis buffer [13], [14].

### Bacterial two-hybrid analysis

Protein-protein interactions were investigated using the BacterioMatch II two-hybrid system (Stratagene), according to the manual [15] with some modifications. The system features a HIS3-*aadA* reporter cassette, whose expression allows *E. coli* growth in the presence of 3-AT (3-amino-1,2,4-triazole), which is a competitive inhibitor of His3 (imidazoleglycerol-phosphate dehydratase), and in the presence of streptomycin.

To test protein-protein interactions, in-frame gene fusions were created in the pBT (bait) or pTRG (target) vectors. PCR primers with suitable restriction sites were constructed and are listed in Table 1. IscS was fused with a bait protein, generating pBT-IscS; DndB-E were fused with target protein, generating pTRG-DptB, pTRG-DptC, pTRG-DptD and pTRG-DptE respectively. The resulting bait and target clones were co-transformed into the reporter strain *E. coli* XL1-Blue MRF′ Kan (Stratagene/Agilent) and selected on LB agar containing 25 µg/ml chloramphenicol (to select for pBT derivatives), 12.5 µg/ml tetracycline (to select for pTRG derivatives), and 50 µg/ml kanamycin (to maintain F′*proAB lacI* ^q^ *ZΔM15* Tn5).

To test for resistance to 3-AT, single colonies were inoculated into 1 mL LB containing the three above antibiotics, and kept shaking overnight at 30°C. 500 µl of this overnight culture was then inoculated into 5 mL SOC medium and incubated for 90 min at 37°C. The cells were then spun down at 3500 rpm for 5 min at room temperature, and the supernatant was carefully removed. The cells were then re-suspended in 2 ml M9^+^ His-drop out broth, collected by centrifugation as described above, and re-suspended in 3 mL M9^+^ His-drop out broth [15]. After incubation for 2 hours at 37°C, three parallel ten-fold dilutions 10^−1^–10^−7^ were prepared and plated 10^0^, 10^−1^, 10^−2^ and 10^−3^ on Selective Screening Medium (SSM) containing 5 mM 3-AT and 10^−4^, 10^−5^, 10^−6^ and 10^−7^ on Nonselective Screening Medium (NSM) without 3-AT. Colonies were counted after 24 h incubation at 37°C. If there were no visible colonies, the plates were incubated in dark at 25°C for another 16 hours.

Putative positive interactions were verified using Dual Selective Screening Medium containing 5 mM 3-AT + 12.5 µg mL^−1^ streptomycin.

### Strep and GST Pull-down

Ten milliliters of *E. coli* BL21 (DE3) strain (harboring Strep-iscS, or GST-DptC, or GST-DptE) was inoculated to 1 L and grow at 37°C for 3 hours with shaking (220 rpm). IPTG was then added to a final concentration of 0.2 mM (from 1000 folds stock). The culture was then moved to 16°C and grew for another 24 hours. The cells were collected by centrifuge for 10 minutes at 4°C.

Cell pellet was re-suspended using Buffer S (25 mM Hepes pH7.6, 100 mM KCl, 10% glycerol, 1 to 10 folds (w/w)) and sonicated for 30 minutes. Cell debris was removed by centrifugation at 15000 g for 20 minutes. Equal volume of the extract was mixed (IscS-_GST_DptC or IscS-_GST_DptE). Two milliliter of the mixture was incubated with 0.1 ml Streptactin resin (Qiagen) or GST resin (Qiagen) pre-equilibrated using Buffer S. After 1 hour, the resin was spin down (400 g, 3 minutes). Supernatant was removed. The resin was wash 5 times using 2 ml Buffer S. The protein was eluted using 0.3 ml Buffer S supplemented with 2.5 mM Desthiobiotin or 20 mM Glutathione. Western blot was done using antibodies from Abcam (ab58626 for GST, or ab76949 for StreptagII).

## Results

### Expression of *S. enterica dptB-E* in *E. coli* BW25113 results in DNA phosphorothioation

Owing to the observation that in *Streptomyces lividans*, *dndA* is essential for DNA phosphorothioation, we sought to find the cysteine desulfurase gene in *E. coli*. The *E.coli* genome was searched for orthologs of a cysteine desulfurase gene.

Fig. 1B shows that there are at least three cysteine desulfurase genes in the *E. coli* genome [10], [11].

Fig. 1C shows that introducing pJTU3510 carrying *dptB-E* four genes, a low-copy plasmid, into *E. coli* BW25113 resulted in DNA S-modification (lane 3). We speculated that *dptB-E*, in cooperation with one or more *E. coli* desulfurase gene, leads to DNA phosphorothioation.

### *Isc*S is responsible for the DNA phosphorothioation *in E.coli*

For DNA phosphorothioation in *E.coli* BW25113, it seemed likely that a protein similar to the cysteine desulfurase DndA was needed in addition to the *S. enterica dptB-E* gene cluster. Individual *E.coli* BW25113 knockout mutants, *ΔiscS*, *ΔsufS* and *ΔcsdA* were available from the Yale Coli Genetic Stock Center. The *iscS* mutant did not survive the transport and was reconstructed (Fig. S1).

*E. coli* BW25113 and the three cysteine desulfurase mutants were transformed with pJTU3510 expressing *dptB-E*, and tested for the phosphorothioation status by Dnd phenotypic assay (DNA smear, an indicator of DNA phosphorothioate modification) (Fig. 2). Only the *iscS* mutant failed to modify its DNA (lane 4), suggesting that only *E. coli* IscS, but not SufS or CsdA, was responsible for DNA phosphorothioation in *E. coli*.

To confirm that *iscS* is responsible for DNA phosphorothioation, *iscS* was cloned into pET15b, and co-transformed with a *dpt* gene cluster harboring low copy number plasmid pJTU3510 into the *E. coli iscS* deletion mutant. Fig. 2 lane 9 shows that DNA phosphorothioation was restored in the strain, proving that IscS, in cooperation with DptB-E, restored DNA phosphorothioation in *E. coli*.

Involvement of IscS in DNA phosphorothioation in *E. coli* was further confirmed by site-directed mutagenesis. Three conserved cysteine residues in IscS, were mutated to Ala, generating three *iscS* cysteine mutants (C111A, C170A, and C328A). These mutants were again co-transformed with pJTU3510 (harboring the *dpt* gene cluster) into the *iscS* deletion mutant. Fig. 2 lane 10–12 shows that only C328A abolished DNA phosphorothioation.

### IscS might participate in DNA phosphorothioation directly

The cysteine desulfurase IscS is a highly conserved master enzyme initiating sulfur transfer via persulfide to a range of acceptor proteins. IscS is involved in various physiological processes, including Fe-S cluster assembly, tRNA modification, and sulfur-containing cofactor biosynthesis. IscS-interacting partners, including IscU, TusA, ThiI, ThiF and MoeB are sulfur acceptors. Other proteins, such as CyaY, IscA and IscX, also bind to IscS, but their functional roles are not directly related to sulfur transfer [16].

**Figure 3:**
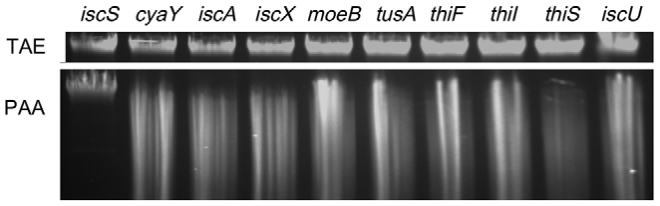
IscS might participate DNA phosphorothioation directly. Ethidium bromide-stained agarose gels. TAE (top gel), samples run in normal TAE buffer; PAA (bottom gel), samples run in TAE containing PAA. Expression of *S. enterica dptB-E* resulted in DNA S-modification and a fluorescent smear in all samples, except for *E. coli ΔiscS*. *IscS* was therefore the only gene that was required for DNA S-modification among the tested deletions.

Mutants of cyaY, iscA, iscU, iscX, moeB, tusA, thiF, thiI and thiS, proteins known to interact with IscS in *E. coli*, were tested for their possibility to participate into DNA phosphorothioation. Fig. 3 shows that none of these genes was required for the modification, as assayed by Dnd phenotype. This suggested that IscS in *E. coli* might participate directly into the modification process.

### Protein-protein interactions between IscS and *Dpt* proteins

The bacterial two-hybrid system was used to detect interactions between *E. coli* IscS and DptB, C, D and E. IscS was fused with the bait protein, while DptB, C, D, and E were fused with the target protein.

**Figure 4:**
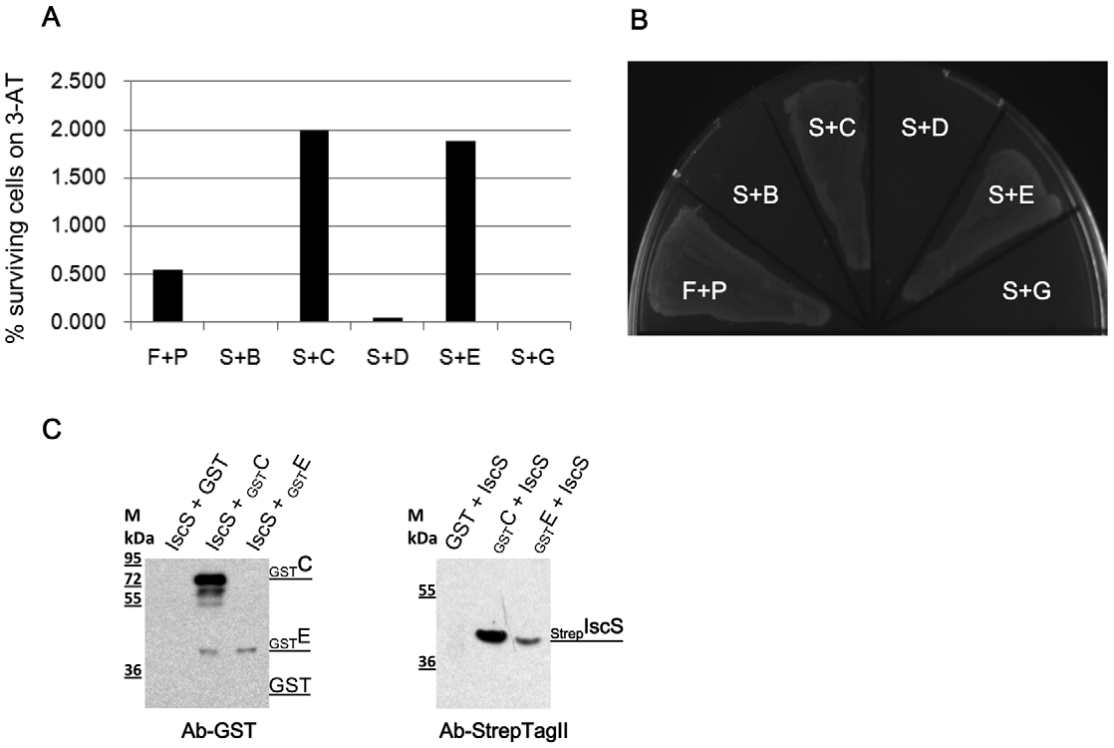
Protein interactions between IscS and Dpt proteins. A.The bar graph shows protein interactions that enable the *E. coli* cells to survive on medium containing 3AT (3-amino-1,2,4-triazole). F, pBT-LGF2; P, pTRG-Gal11P; S, pBT-IscS; B, pTRG-DptB; C, pTRG-DptC; D, pTRG-DptD; E, pTRG-DptE; G, pTRG only. F and P were co-expressed as positive control; S and G were co-expressed as negative control. *E. coli* can grow on 3-AT selective screening medium only when there is a binding interaction between the fusion proteins expressed from the bait and target plasmids. B. Dual selection plate containing 3-amino-1,2,4-triazole and streptomycin. F+P, LGF2+GallP (growth, positive control); S+B, IscS+DptB (no growth, no interaction); S+C, IscS+DptC (growth indicating protein interaction); S+D, IscS+DptD (no growth, no interaction); S+E, IscS+DptE (growth indicating protein interaction); S+G, IscS+pTRG (no growth, negative control). C. Interactions between IscS and DptC as well as IscS and DptE confirmed by pull-down experiments. Left panel: IscS (N terminus Strep tagged) extraction was mixed with _GST_DptC or _GST_DptE extraction and then purified by Streptactin affinity purification. Western blot was done using antibody against GST. Right panel: the mixture was purified by GST affinity purification. Western blotting was done using antibody against StreptagII.

Strong protein-protein interactions were immediately detected between IscS and DptC (2% surviving cells on 3AT), IscS and DptE (2% surviving cells on 3AT), but not between IscS and DptB and DptD (Fig. 4A). These protein interactions were confirmed further by plating the co-transformed strains on medium containing streptomycin (Fig. 4B).

Protein-protein interaction between IscS and DptC as well as IscS and DptE were further confirmed by pull-down experiments. Fig. 4C shows that Strep tagged IscS can pull-down both GST tagged DptC and DptE. Reciprocally, GST tagged DptC and DptE can also pull-down Strep tagged IscS.

## Discussion

IscS is a highly conserved, but functionally versatile pyridoxal-5′-phosphate (PLP)-dependent enzyme. It delivers sulfur to players within various metabolic pathways, including iron-sulfur cluster assembly, thiamine and biotin synthesis, tRNA modifications, and molybdopterin biosynthesis [16], [17]. We show here that IscS can also participate in DNA phosphorothioation.

The involvement of IscS in DNA phosphorothioation could be direct or indirect. By analyzing the Dnd phenotype and the mutants (Fig. 3), we were able to rule out the possibility that IscS participates indirectly via other pathways. We hypothesized that if IscS is involved in the DNA phosphorothioation process directly, we might be able to detect protein-protein interaction between IscS and the Dnd proteins. In keeping with this hypothesis, protein interaction between IscS and DndE and DndC were detected using the bacterial two hybrid system.

There are two potential functions of IscS in the process of DNA phosphorothioation. One is Fe-S cluster assembly for the DndC protein. It is known that DndA can catalyze apo-Fe DndC to its Fe-S cluster form [5]. Another function might be to transfer sulfur from cysteine to the target DNA via protein interactions with the Dnd proteins, which is reminiscent of tRNA modification [18], [19]. These hypothesises are currently under intensive investigation.

## Supporting Information

Figure S1 Disruption of *isc*S gene. A. Replacement of *iscS* by PCR targeting using a *neo* cassette flanked by 50 bp homologous *E. coli* sequences. B. Ethidium bromide-stained agarose gel showing PCR products obtained from *E. coli Δ*iscS and wild-type *E. coli*, using flanking primers.
(TIF)
