# Supplementary figures and images for: A Novel Target of IscS in Escherichia coli: Participating in DNA Phosphorothioation
Source: PLoS One. 2012 Dec 11;7(12):e51265. doi: 10.1371/journal.pone.0051265 (PMC3519819; doi:10.1371/journal.pone.0051265)

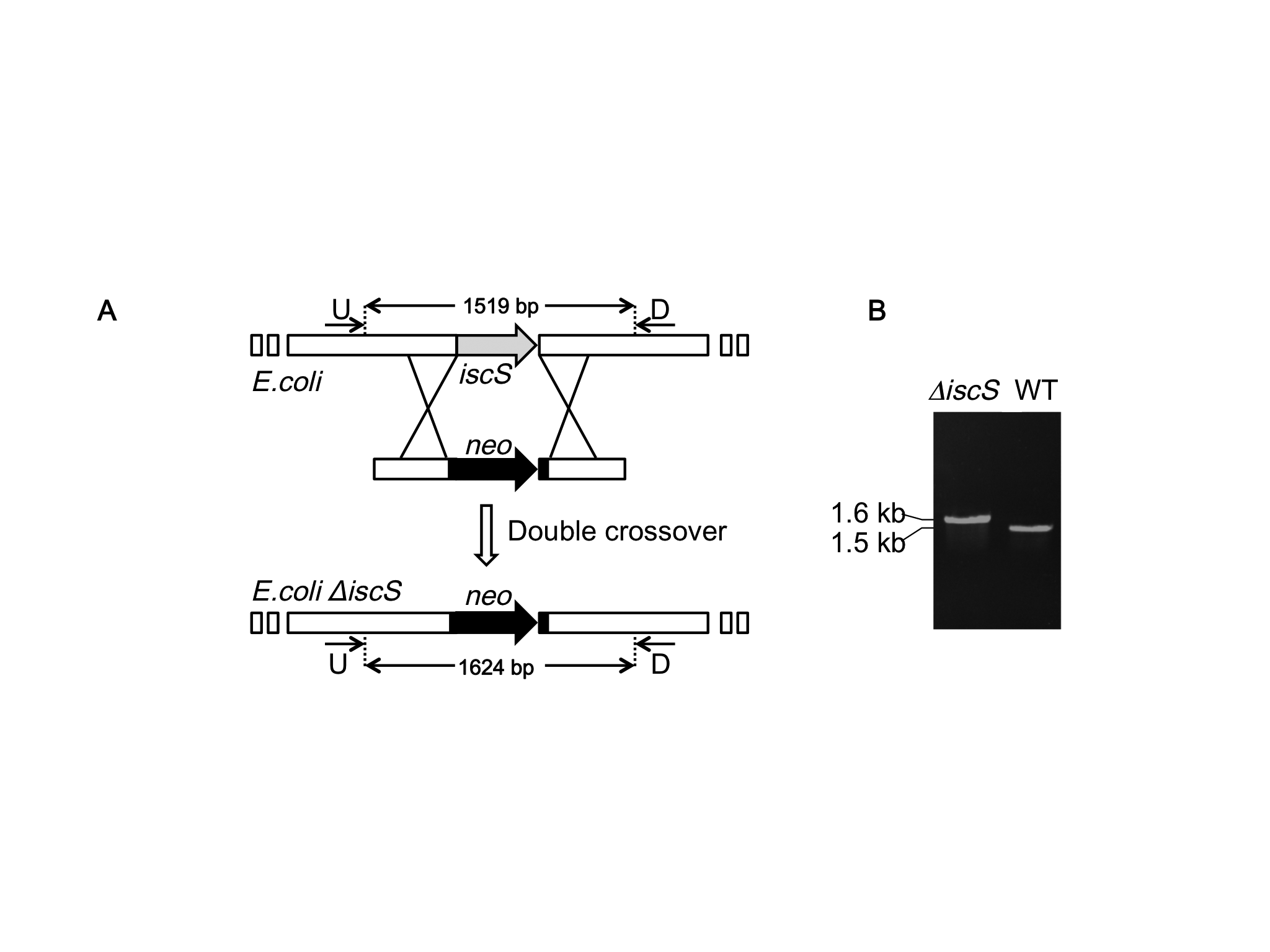

Supplement: Figure S1 — Disruption of iscS gene. A. Replacement of iscS by PCR targeting using a neo cassette flanked by 50 bp homologous E. coli sequences. B. Ethidium bromide-stained agarose gel showing PCR products obtained from E. coli ΔiscS and wild-type E. coli, using flanking primers. (TIF) [file pone.0051265.s001.tif]
